# Atypical Presentation of Cecal Volvulus

**DOI:** 10.7759/cureus.47343

**Published:** 2023-10-19

**Authors:** Nzubechukwu Ijezie, Samson Abolaji

**Affiliations:** 1 Surgery, Dorset County Hospital NHS Foundation Trust, Dorchester, GBR; 2 Surgery, Epsom and St Helier University Hospitals NHS Trust, Sutton, GBR; 3 Medicine, Richmond Gabriel University, Kingstown, VCT

**Keywords:** non-specific abdominal pain, cecal volvulus, cecopexy, acute bowel obstruction, ct -scan

## Abstract

Cecal volvulus, despite being the second most common type of intestinal volvulus after sigmoid volvulus, frequently gets underdiagnosed in clinical practice. Further, management of this intestinal pathology, which requires a considerable amount of expertise, could be lacking due to the similarities in presentation with other intestinal obstructive pathologies, or the infrequency associated with the performance of required diagnostic and therapeutic procedures. We describe a case of a 47-year-old female who presented with acute cecal volvulus. In this case, prompt surgical intervention ensured the best possible outcome with preservation of the cecum via a less sophisticated surgical approach. It therefore becomes apparent that there exists other surgical options in the optimal management of cecal volvulus that ensure no part of the bowel is lost, although this is time-bound.

## Introduction

Cecal volvulus remains an uncommon cause of intestinal obstruction. It accounts for 1-3% of intestinal obstruction and 10-30% of colonic volvulus [1]. Volvulus involves a twist, leading to intestinal luminal occlusion with concomitant pain and distension above the level of occlusion. Volvulus associated with the cecum can take the form of axial-torsion type (type I), loop type (type II), or the much rarer bascule type (type III) [2]. If left untreated, cecal volvulus can lead to intestinal obstruction, distension, ischemia, gangrene, and perforation. Once diagnosed, it is a surgical emergency [1].

## Case presentation

A 47-year-old female, with no major medical history, presented to the hospital with a two-hour history of pain in the abdomen that started centrally and then migrated to the right iliac fossa. The pain, which was noted to come in waves, was associated with nausea and shivering but not vomiting, constipation, or diarrhea.

**Figure 1:**
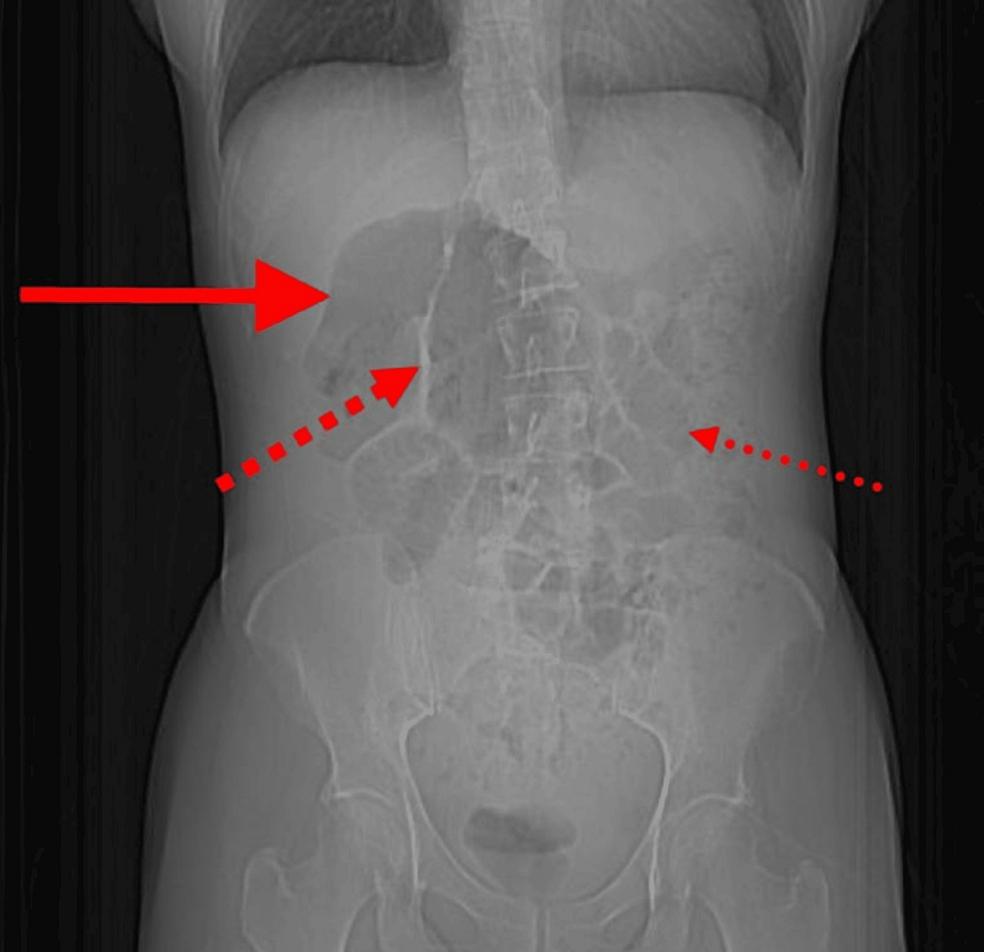
Scout CT scan image showing coffee bean sign (solid red line), split-wall sign (squared dot line), and oedematous bowel wall (round dot line).

Apart from the respiratory rate, which was 22 cycles per minute, the other vital signs were within normal limits, suggesting the former could have been due to the pain, which was rated 6 out of 10 on the numerical rating scale. Physical examination revealed a bloated abdomen that produced tympanic sounds on percussion. Rosving’s sign, rebound tenderness, and Obturator sign were all noted to be positive. A quick bedside ultrasound suggested the absence of free fluid in the abdomen. C-reactive protein (CRP) and white cell count were within normal limits. Computed tomography (CT) scan of the abdomen showed massive bowel dilation around the right upper quadrant (RUQ). Other findings on the CT were coffee bean sign, split-wall sign, and oedematous small bowel wall (Figure 1).

**Figure 2:**
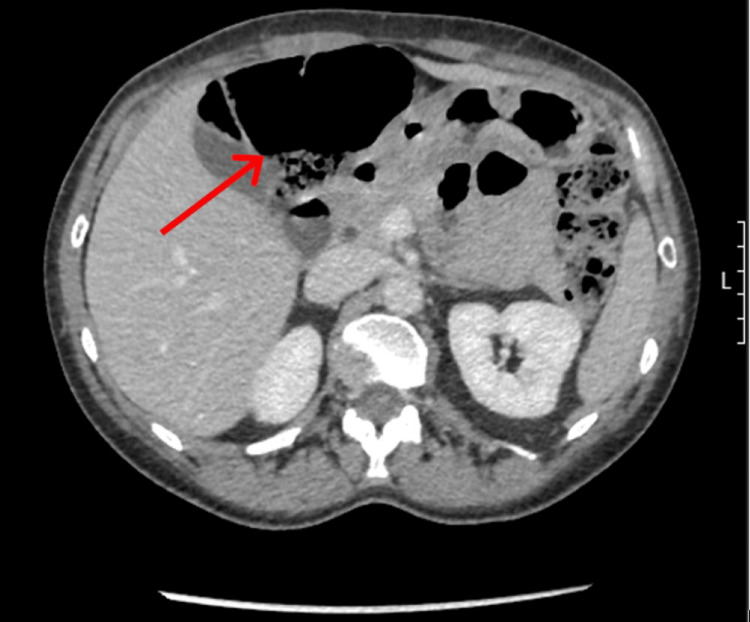
Transverse CT image of massively dilated bowel (solid arrow).

CT was initially reported as acute appendicitis although a note was made of the massive dilation to about 14 mm around the RUQ, suspected to be the appendix (Figure 2).

The patient underwent diagnostic laparoscopy with confirmation of cecal volvulus (270-degree twist) and was consequently treated with cecopexy after inflammation, bruising, and gangrene were ruled out and the integrity of the cecal wall was confirmed. She was discharged the following day. Her recovery was uneventful. At the two-month outpatient follow-up, the patient was devoid of symptoms.

## Discussion

Cecal volvulus is a condition encountered infrequently and makes up 1-3% of intestinal obstruction cases in adults [1].

Clinical presentation of patients with cecal volvulus, as in this case, involves the classic signs of intestinal obstruction such as abdominal pain, distension, ischemia/gangrene, and perforation if left untreated. As such, it is important to make timely diagnosis as delay could be catastrophic. For this reason, abdominal CT is the most preferred modality for timely diagnosis [3].

Crucial in this case, although this patient falls within the expected age range, her major presenting symptom was pain in addition to clinical features suggestive of acute appendicitis. It is important to maintain a high index of suspicion, as if missed, could lead to perforation and sepsis.

Laboratory findings in cecal volvulus are unremarkable and are neither sensitive nor specific for diagnosis. Also, plain abdominal radiographs reportedly carry a poor diagnostic utility as the most commonly visualized abnormalities such as a coffee bean sign directed toward the left upper abdomen, laterally located small bowel swelling compared to the cecum, and absence of gas in the distal colon [4] are non-specific owing to the fact that cecal obstruction, like most other causes of intestinal obstruction, would present similarly [5]. Due to diagnostic dilemma posed by relying on laboratory and plain film radiographs, other modalities that could provide accurate diagnosis need to be used. These include barium enema, CT, colonoscopy, or celiotomy [5].

On CT, the coffee bean sign may be observed [1]. Other findings on CT suggestive of cecal volvulus include marked cecal dilation, whirl sign which is characterized by twisted loops of cecum encircling mesenteric vessels, ileocecal twist where the ileum is caught in the twist, split-wall sign depicted by the apparent split of invaginated cecal walls interposed by pericolic fat, and X-marks-the-spot sign denoting complete winding of twisted bowel loop limbs on each other [6]. The whirl, ileocolic, X-marks-the-spot, and split-wall signs are highly specific for cecal volvulus [6]. In addition, some connected findings such as decompression of the sigmoid colon coupled with evidence of closed loop obstruction on CT could be utilized for rapid diagnosis cecal volvulus.

In very limited cases, barium enema and colonoscopy could be used to both diagnose and treat cecal volvulus. Conventionally, barium enema was the modality of choice investigating cecal volvulus with the “beak sign” confirming the diagnosis, in addition to enabling visualization of the distal colon for the exclusion of other potential pathologies contributing to the formation of volvulus [7]. Nonetheless, owing to the time requirement of the procedure, coupled with potential leakage of contrast in cases of gangrenous/perforated bowel, use of barium enema in diagnosing acute cecal volvulus has heavily declined [8]. Further, the usefulness of colonoscopy in managing acute cecal volvulus is limited given the low success rate [9], in addition to the potential for perforation and delays in definitive treatment following unsuccessful attempts at reduction [1,8].

Surgery is the mainstay of treatment. Although resection offers the least chance of recurrence, cecopexy and cecostomy are other options that could be utilized based on the clinical situation and the overall physiology of the patient. Cecopexy alone is associated with high recurrence rates [10], whereas cecostomy is associated with low recurrence rates but higher rates of morbidity and mortality compared to cecopexy [1]. Although there is paucity of evidence to demonstrate its efficacy, the patient in this case was successfully treated with cecopexy.

## Conclusions

The presentation of cecal volvulus in clinical practice can be non-specific owing to overlap with other forms of intestinal obstruction and, as was noted in this case, acute appendicitis. However, there is a need to have a high index of suspicion as imaging modalities are not foolproof and failure to do so can be catastrophic. As such, direct visualization of the cecum through surgical means provides the surest means of diagnosis and, if promptly done, as in this case, can be successfully managed with cecopexy.
